# Multi-Omics Revealed Resveratrol and β-Hydroxy-β-methyl Butyric Acid Alone or in Combination Improved the Jejunal Function in Tibetan Sheep

**DOI:** 10.3390/antiox13080892

**Published:** 2024-07-24

**Authors:** Qiurong Ji, Fengshuo Zhang, Yu Zhang, Quyangangmao Su, Tingli He, Shengzhen Hou, Linsheng Gui

**Affiliations:** College of Agriculture and Animal Husbandry, Qinghai University, Xining 810016, China; ys220951330640@qhu.edu.cn (Q.J.); ys210951330582@qhu.edu.cn (F.Z.); ys230951330702@qhu.edu.cn (Y.Z.); ys220951330638@qhu.edu.cn (Q.S.); ys220951330641@qhu.edu.cn (T.H.); 1987990009@qhu.edu.cn (S.H.)

**Keywords:** resveratrol, β-hydroxy-β-methyl butyric acid, microbiota, metabolic profiles, Tibetan sheep

## Abstract

Previous research studies confirmed that both resveratrol (RES) and β-hydroxy-β-methyl butyric acid (HMB) improved growth performance by altering intestinal microbiota. However, the mechanism underlying of RES and HMB on intestinal function remains unclear in ruminant. In this study, supplements of RES and HMB alone or in combination were evaluated as promoters of antioxidant capacity, immune response and barrier function, and modulators of the microbiota and metabolite profiles in the jejunum of Tibetan sheep. A total of 120 two-month-old Tibetan rams were randomly divided into four treatments (*n* = 30 per treatment), which were supplemented with a basal diet with 1.5 g RES/d (RES group), 1.25 g HMB/d (HMB group), 1.5 g RES/d plus 1.25 g HMB/d (RES-HMB group), and without additions (Control group). The results showed that RES and HMB improved the antioxidant capacity (CAT, GSH-Px, SOD, and T-AOC), immunity (IgA, IgG, and IgM), and digestive enzyme activity (α-amylase, lipase, and chymotrypsin) of the experimental lambs (*p* < 0.05). Additionally, jejunal morphology including villus width, villus height, and muscle layer thickness exhibited a significant difference when rams were fed diets supplemented with RES and HMB (*p* < 0.05). Furthermore, the determination of fermentation parameters showed that the butyrate concentration in the RES-HMB group was greater than those in the C and RES groups (*p* < 0.05). When compared to the C group, barrier-related gene expression (*MUC-2*, *ZO-1*, and *IL-10*) was significantly increased in the RES-HMB group (*p* < 0.05). Dietary RES and (or) HMB supplementation significantly increased the abundance of *Methanobrevibacter*, *Actinobacteriota* and *Bacillus* (*p* < 0.05). The abundance of differential bacteria was positively associated with butyrate concentration (*p* < 0.05). Metabolome analysis revealed that alpha ketoglutarate, succinic semialdehyde, and diacetyl as well as butanoate metabolism pathways connected to the improvements in butyrate concentration by RES and (or) HMB supplementation. Collectively, our results suggested that RES and (or) HMB supplementation improved butyrate concentration via regulating the microbial community (*Methanobrevibacter*, *Actinobacteriota* and *Bacillus*) and metabolism (alpha ketoglutarate, succinic semialdehyde, and diacetyl), thus contributing to jejunal morphology, antioxidant capacity, immune response, digestive enzyme activity, and barrier function.

## 1. Introduction

As a selectively permeable barrier, the intestine serves the absorption of nutrients including organic acids, electrolytes, and water, as well as protecting against food-borne pathogens [1]. Within the intestinal tract of mammals exists abundant and diverse communities of symbiotic microbes [2]. The gut microbiome is a widely explored diverse population and participates in maintaining metabolism, stabilizing immune function, and resisting pathogens in the health of the host [3]. Research has demonstrated that regulating animal feed can affect the composition, abundance, and metabolites of gut microbiota, thereby manipulating animal health [4]. Therefore, research into substitutes such as lysozyme, essential oils, bacteriophages, and prebiotics is crucial [5].

Resveratrol (RES) is a polyphenolic plant antitoxin produced by plants in response to environmental stress, present in at least 72 plants, commonly found in grapes, peanuts, and berries [6]. This plant antitoxin exhibits a wide range of biological properties such as antioxidant [7], anti-saccharification [8], anti-aging [9], anti-inflammatory [10], anti-cancer [11], and neuroprotective properties [12]. Previous studies indicated that the gut microbiota maintained redox homeostasis by regulating the production of reactive oxygen species (ROS) and RES served as a scavenger for scavenging free radicals such as ROS. Given the bidirectional relationship between the two terms, it is hypothesized that the gut microbiota may serve as a target for resveratrol to maintain intestinal homeostasis [13]. In an early study of a dextran sodium sulfate (DSS) rat colitis model, adding RES to the diet repaired the colon barrier and reversed the ecological imbalance of microorganisms [14]. Upon arterial remodeling, RES regulated the microbiota-derived metabolites (e.g., short-chain fatty acids) [15].

β- Hydroxyl group- β- Methyl butyric acid (HMB) is an organic acid containing five carbons, which is produced by leucine [16], and promotes protein synthesis [17]. According to the report, 0.10% HMB supplementation altered the abundance, diversity, and composition of gut microbiota in broilers, thereby inhibiting liver fat deposition [18]. Studies have also shown that as a probiotic formulation, HMB can utilize its mechanism to reprogram gut microbiota and metabolism to reverse HFD-induced obesity [19]. However, the endogenous production of HMB is very low, and the amount that can be converted into HMB from the normal intake of leucine does not exceed 10%. Therefore, it is necessary to supplement HMB with a diet to fully exert its effects [20].

However, there have not been reports on the study of the dietary supplementation of RES and HMB in ruminant. It is hypothesized that RES and HMB could have a positive effect on the microbiota and metabolome, thereby supporting intestinal function. Therefore, the aims of present study to explore the mechanism of RES and HMB alone or in combination on the jejunal function using 16s RNA gene sequencing and non-targeted metabolomics in Tibetan sheep.

## 2. Materials and Methods

### 2.1. Ethical Statement

The protocol and methodology of the present study were approved by the Institution of Animal Care and Use Committee at Qinghai University, China (Xining, China; Permit No. QUA-2020-0709).

### 2.2. Experimental Design

The Tibetan rams were selected from Qinghai Xiangkameiduo animal husbandry Co., Ltd. (Gonghe, Qinghai province, China). All individuals were immunized by a standardized procedure (e.g., ovine braxy, struck, lamb dysentery, enterotoxemia, and ovinia) and weaned at 60 d of age. In this experiment, one hundred and twenty Tibetan rams aged 2 months old (initial weight: 15.5 ± 0.14 kg) were clinically evaluated and were apparently healthy, and they were randomly divided into 4 treatments optionally. The four treatments were supplemented with a basal diet with 1.5 g RES/d (RES group), 1.25 g HMB/d (HMB group), 1.5 g RES/d plus 1.25 g HMB/d (RES-HMB group), and without additions (Control group), respectively. Each treatment consisted of 5 replicate pens with 6 rams. The feeding trial continued for 100 days, including a 10-day acclimation period and a 90-day experimental period. All of the sheep had access to fresh water ad libitum. The total mixed ratio contained 70% concentration and 30% forage (dry matter basis). The diet composition and nutrient levels are listed in Table 1.

### 2.3. Sample Collection

At the end of the experiment, twenty-four Tibetan lambs (*n* = 6 per treatment) were slaughtered at a commercial slaughterhouse. The jejunal contents were collected and stored at −80 °C for omics analysis. Synchronously, the jejunal tissues were collected and fixed with 4% paraformaldehyde for histological analysis.

### 2.4. Enzyme-Linked Immunosorbent Assay (ELISA)

The jejunal contents were centrifuged (2500× *g*) for 15 min at 4 °C. The indicators of supernatant including antioxidant capacity, immune response, and digestive enzyme activity were determined using ELISA kits (Jiangsu Meimian Industrial, Yancheng, Jiangsu, China).

### 2.5. Jejunal Morphology

The fixed jejunal tissues were embedded in paraffin and cut to 3 µm. Five discontinuous sections of each sample were selected for hematoxylin–eosin staining. Stained sections were observed using an Olympus BX51 microscope. The villus height, villus width, mucosal thickness, muscularis propria thickness, crypt depth, and villi height/crypt depth (VH/CD) were measured by Imagingeproplus 6.0 analysis software.

### 2.6. Quantitative PCR (qPCR)

The total RNA of jejunal tissues was extracted using Transzol Up (TRAN, Beijing, China) and reverse transcribed using the Universal SYBR Green qPCR Mix kit (Azaood, Beijing, China). The qPCR reaction procedure followed Zhu et al.’s (2024) method [21]. The relative expression of the gene was calculated using the 2^−ΔΔCt^ method. Information on the primer sequences is shown in Table S1.

### 2.7. Short-Chain Fatty Acid (SCFC) Composition

The concentration of SCFCs was detected by gas chromatography–mass spectrometry (7890B GC System, Aglient, Billerica, MA, USA) with an Agilent DB-FFAP capillary column (30 m × 250 μm × 0.25 μm) to separate the samples. The initial temperature was raised form 90 °C to 160 °C at a rate of 10 °C/min. Then, 40 °C temperature was raised to 160 °C and maintained for 5 min. The carrier gas used was helium, with a flow rate of 1.0 mL/min.

### 2.8. 16S rDNA Sequencing

The genomic DNA of jejunal contents was extracted using the HiPure Stool DNA kits (Magen, Guangzhou, China). The 16S rRNA genes targeted the V3-V4 hypervariable region and were amplified using the 341F-806R primers (5′CCTACGGGNGGCWGCAG3′) and 806R (5-GGACTACHVGGGTWTCTAAT-3′). The paired-end sequencing was accomplished using an Illumina MiSeq PE250 platform (Illumina Inc., San Diego, CA, USA). To obtain the high-quality clean tags, raw reads were filtered using the FASTP (Version 0.18.0). The abundance statistics of taxonomy was visualized using Krona (Version 2.6). Alpha diversity analysis was calculated in QIIME (Version 1.9.1). Principal coordinate analysis (PCoA) analysis was conducted using the package “vegan” in R software (Version 4.3.1).

### 2.9. Metabolome Sequencing

Non-targeted metabolomics was performed using an UHPLC system (1290 Infinity LC, Agilent Technologies) coupled with a quadrupole time of flight (AB Sciex TripleTOF 6600) in Shanghai Applied Protein Technology Co., Ltd. Chromatography was carried out with an ACQUITY UPLC BEH column (2.1 × 100 mm, 1.7 μm) (Waters, Ireland). Raw MS data were converted into mzXML format using ProteoWizard MSConvert (https://proteowizard.sourceforge.io/download.html, accessed on 7 November 2023). Orthogonal partial least squares discriminant analysis (OPLS-DA) was performed by the “ropls” package in R (Version 3.3.2). The differential metabolites were defined by variable importance projection (VIP) values above 1.0 and *p* < 0.05. The identified metabolites were mapped to the Kyoto Encyclopedia of Genes and Genomes (KEGG) Pathway database (https://www.kegg.jp, accessed on 23 November 2023).

### 2.10. Statistical Analysis

The results of jejunal morphology, antioxidant capacity, immune response, digestive enzyme data, relative gene expression, and SCFAs were analyzed using a general linear model (GLM, SPSS 22.0). Results were presented as means ± SEM. A *p*-value of <0.05 was considered to have a significant correlation. The correlation heat maps were drawn using the R package (Version 3.3.2).

## 3. Results

### 3.1. Antioxidant Capacity, Immune Response, and Digestive Enzyme Activity of Jejunal Contents

**Figure 1:**
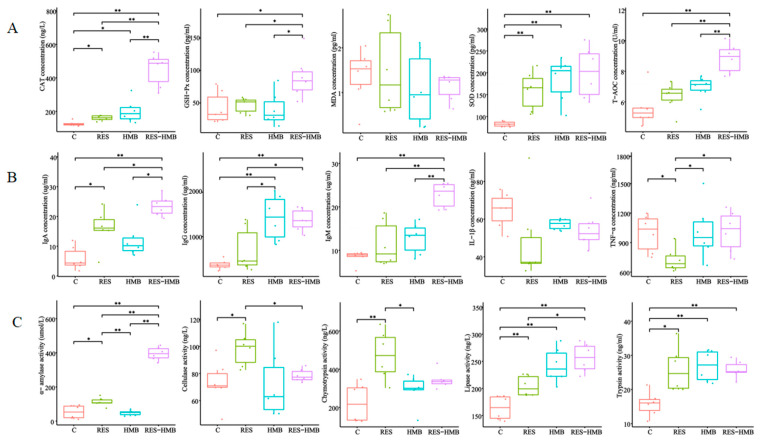
Effects of supplementation with RES and HMB alone or in combination on the anti-oxidative parameters (**A**), immune status (**B**) and digestive enzyme activity (**C**) in jejunal contents. C, a basal diet; RES, a basal diet plus 1.5 g/head/d resveratrol; HMB, a basal diet with 1.25 g/head/d β-hydroxy-β-methyl butyrate; RES-HMB, a basal diet supplemented with 1.5 g/head/d resveratrol and 1.25 g/head/d β-hydroxy-β-methyl butyrate. * *p* < 0.05 and ** *p* < 0.01.

For the antioxidant capacity (Figure 1A), the RES-HMB group showed higher concentrations of catalase (CAT, *p* < 0.01), glutathione peroxidase (GSH-Px, *p* < 0.05), and superoxide dismutase (SOD, *p* < 0.01) compared to the C group. For the immune response (Figure 1B), dietary RES and (or) HMB supplementation significantly improved the levels of immunoglobulin (Ig) A, IgG, IgM, and tumor necrosis factor-α (TNF-α) compared to the C group (*p* < 0.01). The combination of RES with HMB exhibited an optimal effect. For digestive enzyme activity (Figure 1C), the contents of α-amylase and lipase in the RES-HMB group were greater than the C and RES groups (*p* < 0.05 or *p* < 0.01).

### 3.2. Jejunal Morphology

**Figure 2:**
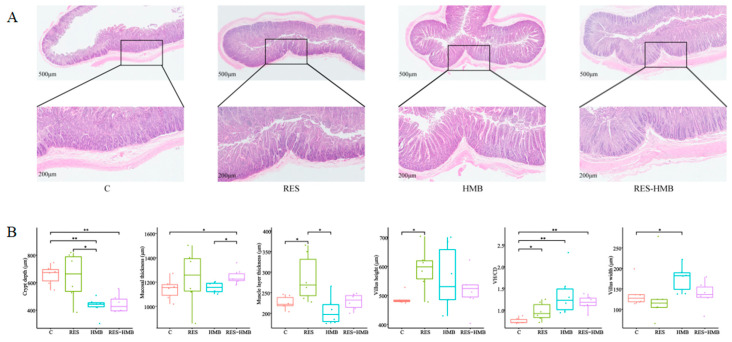
Effect of RES and HMB alone or in combination on jejunal morphology in Tibetan sheep. (**A**) Representative histological images of jejunal slide stained with hematoxylin–eosin (original magnification 500× and 200× μm). (**B**) Box plot of jejunal measurement indicators. VH/CD: villus height/crypt depth. * *p* < 0.05. ** *p* < 0.01.

H&E sections revealed the morphological alternations in the jejunal tissue of Tibetan sheep (Figure 2A). The mucosal thickness (*p* < 0.05) and VH/CD (*p* < 0.01) in the RES-HMB group was significantly increased compared with the C group. Compared to the C group, the RES group exhibited a significant increase in villi height and muscle layer thickness (*p* < 0.05). Additionally, the crypt depth of the C group was significantly increased compared with the HMB and RES-HMB groups (*p* < 0.01) (Figure 2B).

### 3.3. Jejunal Barrier-Related Genes Expression

**Figure 3:**
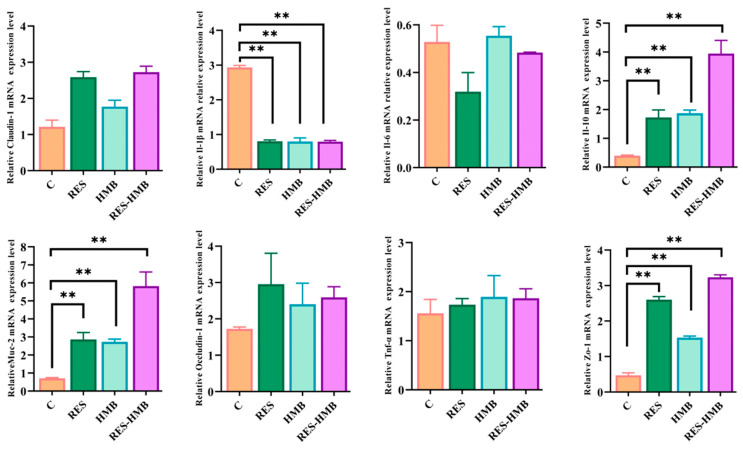
Effects of supplementation with RES and HMB alone or in combination on jejunal barrier of Tibetan sheep. ** *p* < 0.01.

The barrier-related genes expression were identified in jejunum tissues (Figure 3). The supplementation of RES and HMB alone or in combination significantly increased the mRNA expression of interleukin-10 (*IL-10*), mucin 2 (*MUC-2*), and zonula occludens-1 (*ZO-1*) (*p* < 0.01), whereas it decreased the mRNA expression of *IL-1β* (*p* < 0.01).

### 3.4. SCFA Concentration

As shown in Table 2, the concentrations of butyric acid were significantly increased in the RES-HMB group when compared to the C and HMB groups (*p* < 0.05). However, no significant difference was observed in the concentrations of other SCFAs among the four treatments (*p* > 0.05).

### 3.5. Bacterial Community Composition Analyses

**Figure 4:**
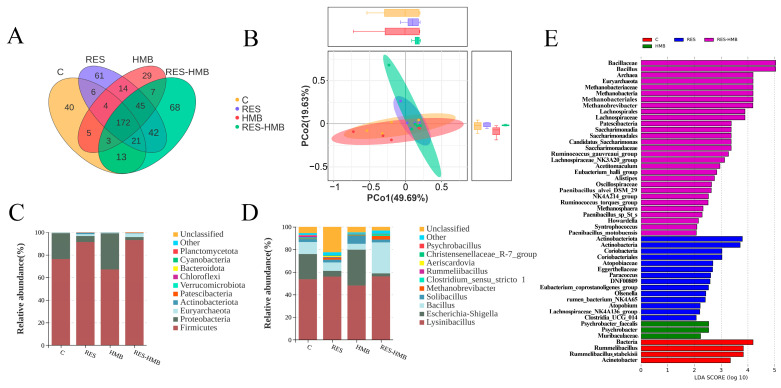
OTU Venn diagram of the overlap of jejunal microbiota (**A**). Principal coordinate analysis (PCoA) of bacterial communities and Anosim analysisin the jejunal contents of Tibetan sheep (**B**). Relative abundance of bacteria community proportion at the phylum (**C**) and genus (**D**) levels, as analyzed by the LDA effect size (LEfSe) method (**E**).

A total of 530 OTUs, including common and unique, were identified as showed in Figure 4A. Of these, 332 OTUs belonged to the common type, and there were 40 unique OTUs in the C group, 61 unique OTUs in the RES group, 29 unique OTUs in the HMB group, and 68 unique OTUs in the RES-HMB group. When examining the community structure (Table 3), no significant differences in the Chao1, Ace, Shannon, and Simpson of jejunal bacteria among the four treatments were observed. The result of the PcoA analysis showed distinct differences among the treatments (Figure 4B). The *Firmicutes*, *Proteobacteria*, and *Actinobacteria* were the dominant phyla of the jejunal content, accounting for 82.00%, 15.72% and 1.48% (Figure 4C). At the genus level (Figure 4D), the predominantly abundant ones were *Lysinibacillus* (53.33%), *Escherichia-Shigella* (15.49%), and *Bacillus* (12.66%). LEfSe analysis identified eight genus and three phylum that were enriched in the four treatments (Figure 4E). The *Patescibacteria*, *Euryarchaeota*, *Bacillus*, *Lachnospiraceae_NK3A20_group*, *Alistipes*, *Ruminococcus_gauvreauii_group*, *Methanobrevibacter*, and *Candidatus_Saccharimonas* were significantly enriched in the RES-HMB group.

**Figure 5:**
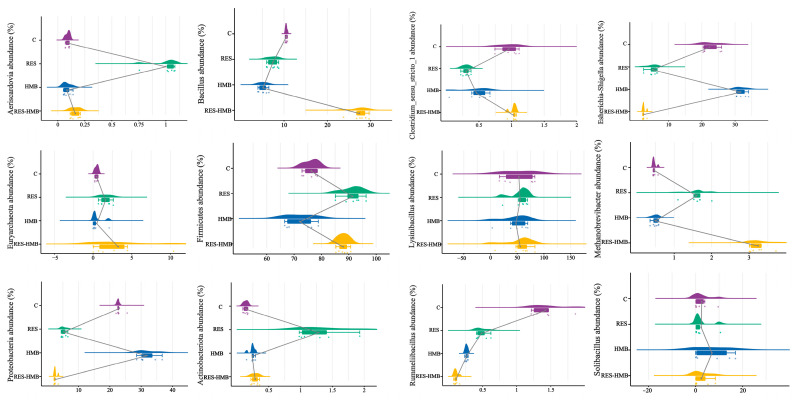
Cloud and rain plots showing the bacterial community compositions in the jejunal content fractions of the C, RES, HMB, RES and HMB treatments at the phylum and genus levels. The gray line represents the average value of each group. Purple represents C group, green represents RES group, blue represents HMB group, and yellow represents RES-HMB group.

As seen via composition analysis (Figure 5), the relative abundance of *Aeriscardovia*, *Firmicutes*, *Bacillus*, *Methanobrevibacter*, and *Actinobacteriota* was significantly increased in the RES-HMB group compared with the C group. For the RES group, the relative abundance of *Euryarchaeota*, *Firmicutes*, *Methanobrevibacter*, *Actinobacteriota*, and *Aeriscardovia* was significantly increased compared to the C group. For the HMB group, the relative abundance of *Actinobacteriota*, *Proteobacteria*, and *Escherichia-Shigella* was significantly increased compared to the C group. Overall, the supplementation of RES and HMB alone or in combination significantly increased the relative abundance of *Actinobacteriota*.

### 3.6. Metabolite Profiles

**Figure 6:**
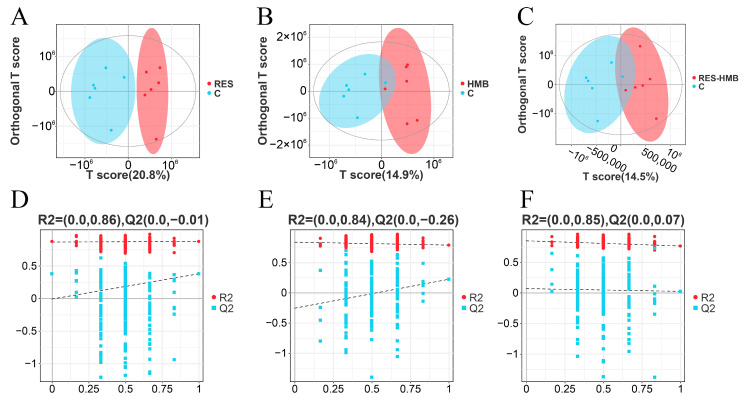
OPLS-DA score plots for C and RES groups (**A**), C and HMB groups (**B**), C and RES-HMB groups (**C**). Permutations test of PLS-DA for C and RES groups (**D**), C and HMB groups (**E**), C and RES-HMB groups (**F**).

The OPLS-DA analysis displayed a distinct separation between the inter-groups (Figure 6A–C). The diagrams of the permutation test of PLS-DA showed that the values of R2X, R2Y, and Q2 were 0.000, 0.860, and −0.010 (RES Vs. C, Figure 6D), 0.000, 0.840, and −0.260 (HMB Vs. C, Figure 6E), and 0.000, 0.850, and 0.070 (RES-HMB Vs. C, Figure 6F), which demonstrated that different dietary supplements in these treatments influenced the metabolic pathways of the Tibetan sheep.

**Figure 7:**
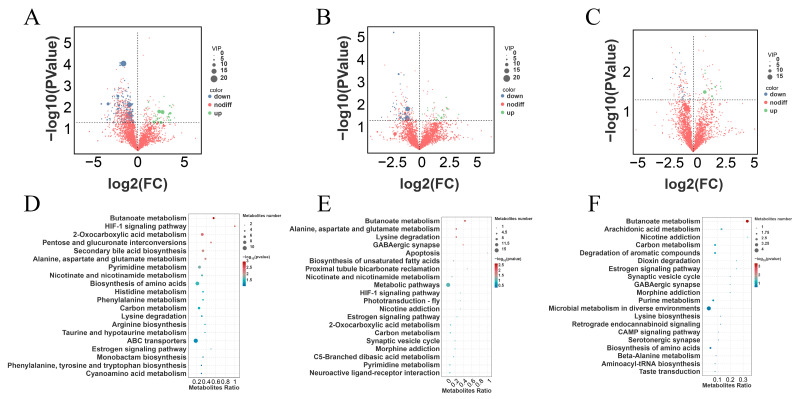
Volcano plot of differential metabolites in C and RES groups (**A**), C and HMB groups (**B**), C and RES-HMB groups (**C**). KEGG pathway enrichment of differential metabolites in C and RES groups (**D**), C and HMB groups (**E**), C and RES-HMB groups (**F**). up: significantly up-regulated metabolites; nodiff: no significantly different metabolites; down: significantly down-regulated metabolites.

According to the analysis of differential metabolites (DMs), a total of 300 DMs were identified (VIP > 1 and *p* < 0.05). Of these, 194 DMs (39 up-regulated and 155 down-regulated) were observed in the RES and C group (Figure 7A). Moreover, 65 DMs (20 up-regulated and 45 down-regulated) were observed in the HMB and C groups (Figure 7B). Additionally, 41 DMs (14 up-regulated and 27 down-regulated) were observed in the RES-HMB and C groups (Figure 7C).

A total of eight common DMs including Ala-Asp, His-Lys, 2′,6′-dihydroxy-4′, 4-dimethoxychalcone, 1-oleoyl-2-myristoyl-sn-glycero-3-phosphocholine, Pg 36:2, 1,2-dioleoyl-sn-glycero-3-phosphoethanolamine, gamma-aminobutyric acid and diacetyl were observed in these four treatments.

Metabolic pathway enrichment analyses showed that the DMs in the C and RES groups were mainly enriched in the “Biosynthesis of amino acids”, “ABC transporterse”, “2-Oxocarboxylic acid”, “Butanoate metabolism”, and “Secondary bile acid biosynthesis” (Figure 7D). The DMs in the C and HMB groups were mainly enriched in “Metabolic pathways”, “Butanoate metabolism”, and “Lysine degradation” (Figure 7E). The DMs in the C and RES-HMB groups were mainly enriched in “Microbial metabolism in diverse environments”, “Butanoate metabolism”, and “Carbon metabolism” (Figure 7F).

### 3.7. Correlation Analysis

**Figure 8:**
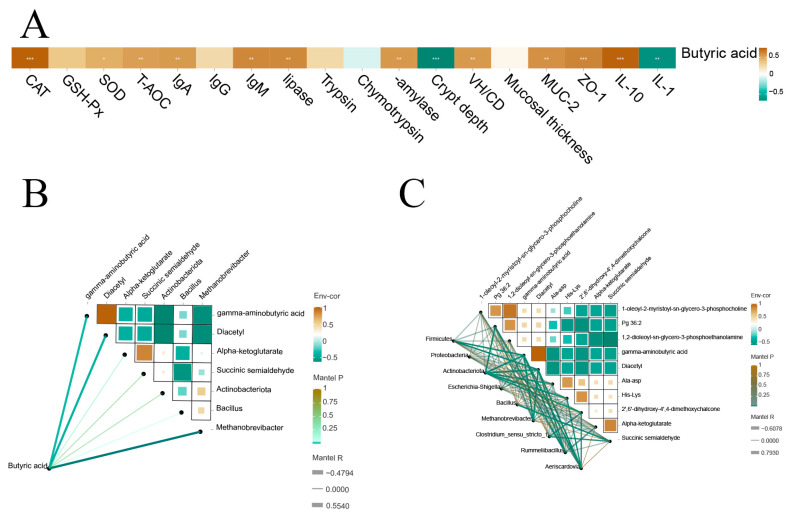
The correlation heat maps between butyrate and anti-oxidative parameters, immune status, digestive enzyme activity, jejunal morphology, jejunal barrier (**A**). Spearman-related heatmap of butyrate and bacteria and metabolites in jejunum of Tibetan sheep (**B**). Spearman correlation heatmap between jejunal microbiota and metabolome (**C**). * *p* < 0.05. ** *p* < 0.01. *** *p* < 0.01.

As showed Figure 8A, the concentration of butyric acid was positively related to the antioxidant capacity (GSH-Px, T-AOC, CAT, and SOD), immune response (IgG, IgM, and IgA), digestive enzyme activity (trypsin, α-amylase, and lipase), morphological indicators (VH/CD), and barrier-related genes expression (*IL-10*, *MUC-2*, and *ZO-1*), while it was negatively correlated with crypt depth and the expression of *IL-1β*.

Additionally, the butyric acid concentration was positively related to the abundance of *Aeriscardovia*, *Actinobacteriota*, and *Methanobrevibacter*, while it was negatively correlated with the contents of diacetyl (Figure 8B).

A correlation network was carried out to reflect the correlation between differential bacteria and differential metabolites (Figure 8C). The abundance of *Aeriscardovia* was positively correlated with 2′,6′-dihydroxy-4′,4-dimethoxychalcone and His-Lys. Furthermore, the abundance of *Actinobacteriota* was positively correlated with 2′,6′-dihydroxy-4′,4-dimethoxychalcone, Ala-Asp, and His-lys, while it was negatively correlated with gamma-aminobutyric acid, diacetyl, and Pg 36:2. The abundance of *Escherichia-Shigella* was positively correlated with gamma-aminobutyric acid, and diacetyl.

## 4. Discussion

It was pointed out that antioxidant enzymes constitute the first-line defense of an antioxidant defense system (CAT, GSH-Px, SOD, T-AOC) [18,22,23]. In an investigation, Ding et al. found that dietary RES increased the activities of SOD, GSH-Px, and T-AOC in the jejunum of laying hens [24]. Yang et al. also identified that dietary supplementation with 400 mg/kg RES alleviated oxidative injury by improving the activity of SOD in the duck jejunum [25]. Similarly, supplementing HMB increased the number of CD3+ and CD8+ cells and reduced the pro-inflammatory cytokine levels [26,27]. As a type of polyphenol, RES exhibited antioxidant properties similar to other polyphenols [28]. HMB is a derivative of the essential amino acid leucine metabolite a-ketoisohexanoic acid, which activates antioxidant stress via mediating ketoisohexanoic acid and isovaleryl CoA [29,30]. Our results indicated that both the RES and HMB groups significantly increased the activities of CAT and SOD in the jejunal tissues, while the RES-HMB group significantly increased the concentration of CAT, GSH-Px, SOD, and T-AOC. It is speculated that the combination of RES and HMB could enhance antioxidant enzyme activity through eliminating excess reactive oxygen (ROS) in the jejunum.

Previous research has established that RES supplementation in weaned piglets’ diets increased the concentrations of IgA and IgM in the jejunum [31]. Similarly, the levels of IgA, IgG, and IgM in piglets were increased to varying degrees with different doses of RES supplementation [32]. Moreover, a study reported RES alleviated the equine metabolic syndrome of horse via reducing the concentrations of TNF-α [33]. Evidence suggested that HMB reduced inflammatory cytokine production, thereby regulating excessive inflammation [34,35]. Using 0.2% functional amino acids (AAs) reduced weaning-induced stress and inflammation in the early stages of the growth of pig [36]. Additionally, functional AAs and polyphenols reduced dietary corticosterone-induced intestinal damage [37]. In this study, the concentrations of IgA, IgG, and IgM were significantly increased in the RES-HMB group. We speculated that the complex effect of RES and HMB regulated the immune status of the jejunum by interfering with immune cell regulation and the synthesis of pro-inflammatory cytokines.

In current study, cellulase and chymotrypsin in the contents of the jejunum were increased in the RES group. Moreover, α-amylase, lipase, and trypsin activities increased in all experimental groups, while there were more remarkable effects in the RES-HMB group. It was reported that adding 800 mg/kg RES in the diet activated lipase activity in rainbow trout [38]. Other studies have found that RES supplementation improved digestive capacity by enhancing the activity of digestive enzymes in the duodenum of Siberian sturgeon [39]. Foye et al. found that HMB significantly promoted the interaction of digestive enzymes in the jejunum of turkeys, thereby enhancing the digestion and absorption of nutrients [40]. One study found that Hawthorn, rich in acid phenols and amino acids, is endowed with the ability to promote the digestive ability by increasing the activity of digestive enzymes in the small intestine [41]. Therefore, supplementing RES and HMB simultaneously may promote the digestive ability of the jejunum in Tibetan sheep by increasing the digestive enzyme activity in the jejunal contents.

Villus height, villus width, muscle layer thickness, and crypt depth are important indexes for evaluating intestinal integrity and absorptive and digestion capacity [42,43,44]. In the current experiment, the permeability of the jejunum was improved with the RES-HMB supplement. The VH/CD of the jejunum exhibited significant increases in all treatments. The crypt depth demonstrated significant decreases in the HMB and RES-HMB groups. Apart from this, RES treatment significantly increased the muscle layer thickness and villus height of the jejunum. The HMB supplement and RES-HMB supplement significantly increased the villus width and mucosal thickness, respectively. Chen et al. confirmed that dietary RES supplementation contributed to the development of villus height in the jejunum of weaning piglets [45]. Another study reported that adding 400 mg/kg of RES improved jejunal morphology when exposing the heat stress in black bone chickens [46]. In addition, the supplementation of HMB significantly increased the value thickness of the mucosa, height, and thickness of villi in the jejunum of broiler [47]. HMB supplementation in weaned piglets increased villus high in the jejunum while decreasing crypt depth in the ileum [48].

The intestinal morphology, intestinal mucosal epithelium, and intestinal epithelium, among others, constitute the physical barrier of the gut. *MUC-2* plays an important role in mucins by labeling goblet cells in the intestine [49]. As the first epithelial tight junction protein, *ZO-1* is critical for efficient mucosal repair following chemical injury and mucosal healing after immune-mediated injury in intestinal epithelial cells. The abundant immune cells and related lymphoid tissue in intestinal epithelial cells jointly constitute the immune barrier of the intestine. Pro-inflammatory cytokine (*IL-1β*) and anti-inflammatory cytokine (*IL-10*) are essential cytokines for activating the immune system [50]. Prior research indicates that resveratrol treatment not only enhanced the expression of *MUC-2* in mice induced by HFD [51] but also reversed intestinal damage induced by *IL-1β* [52]. A study on the heat-stress-induced impairment of the jejunal mucosa in black-boned chicken reported that RES increased the expression of *ZO-1*. RES supported the mRNA expression of *IL-10* in the jejunal mucosa of weaning piglets [45]. Zhou et al. confirmed that HMB repaired deoxynivalenol-induced intestinal injury by modulating the expression of *MUC-2* [53]. In young grass carp, HMB promoted anti-inflammatory cytokines and down-regulated pro-inflammatory cytokine mRNA levels to execute immunological barrier function in the small intestinal mucosa [54]. There is also a study that indicates that adding AAs and polyphenols to a diet can enhance the physical and immune barrier of the jejunum in broiler chickens [37]. In this experiment, *MUC-2*, *ZO-1*, and *IL-10* were significantly increased, while *IL-1β* decreased by RES and HMB treatments. In general, all results represented here were consistent with those of previous studies, which suggested that the addition of RES and HMB alone or combination to Tibetan sheep diets contributed to jejunal permeability and the jejunal barrier.

As important fuels for intestinal epithelial cells (IECs), SCFAs are the main carbon flux in the diet and the main metabolite produced by the breakdown of the gut microbiota [55]. Previous research showed that RES restored intestinal bacteria to a stable level and increased the production of i-butyric acid in a murine model [56]. Baghbanzadeh-Nobari et al. found that supplementing ewe diets with 2-hydroxy-4 (methylthio) butaoic acid isopropyl ester (HMBi) increased the concentration of total VFA in the rumen [57]. These studies are consistent with our research results showing that RES-HMB treatment significantly increases the level of butyric acid in the jejunum. Although butyric acid cannot be used as a free radical scavenger, it can serve as a secondary antioxidant by affecting the DNA repair system and the levels of enzymatic antioxidants [58]. It can also induce mitochondrial autophagy by activating AMPK, thereby alleviating oxidative stress [59]. As a histone deacetylase inhibitor (HDAC), butyric acid inhibits nuclear factors-κB (NF- κB), thereby down-regulating the expression of *IL-1β*, *IL-6*, and *TNF-α*. Meanwhile, butyric acid acted as a ligand to activate G protein-coupled receptors GPR 41, GPR 43, and GPR 109, promoting the expression of anti-inflammatory factors [60]. In this study, butyric acid was positively related to GSH-Px, SOD, T-AOC, CAT, IgG, IgM, IgA, *IL-10*, *MUC-2*, and *ZO-1*. Therefore, butyric acid fermented with RES and HMB, thereby improving the antioxidant function, immune system, intestinal development, and intestinal barrier.

In this study, the relative abundance of *Actinobacteriota* was increased in the three treatments compared to the C group. As a key modulator in the maintenance of gut barrier homeostasis, *Actinobacteria* regulated the mucin biosynthesis and catabolism [61]. Besides this scenario, *Actinobacteriota* promoted intestinal digestion and absorption [62]. A study related to wild-type mice displayed that *Actinobacteria* interrelated with the SCFA in the cecum [63]. In the present study, the relative abundance of *Methanobrevibacter* and *Bacillus* was increased in the RES-HMB group. *Methanobrevibacter* can be translocated from the ileum and cecum to lymphatic tissues via immune cells, thereby regulating the immune system of pigs [64]. The deficiency of *Methanobrevibacter* disrupted the homeostasis inside the colon, leading to a decrease in mitochondrial SCFA oxidation. The gene encoding NOX was identified in *Methanobrevibacter* smithii to catalyze the oxidation of NADH and convert O_2_ to H_2_O, playing an important role in protecting organisms from oxidative stress and maintaining NAD+/NADH balance [65,66]. It has been observed that *Bacillus* has multiple effects in preventing animal intestinal infections, stimulating the immune system, reducing oxidative stress and breaking down mucins to convert them into short-chain fatty acids [67,68,69]. In our study, butyric acid was positively related to the abundance of *Actinobacteriota*, *Methanobrevibacter*, and *Bacillus*. Therefore, these results indicated that immune response, oxidative stress, digestive enzyme activity, intestinal barrier, and SCFA were associated with intestinal microbial composition in RES and HMB alone or in combination.

We chose pathways and important metabolites with significant differences to describe and explain their relationship with phenotypes and screened differential genera. In the present study, the concentrations of alpha-ketoglutarate and succinic semialdehyde related to butanoate metabolism are increased when fed RES and HMB. Butanoate metabolism is transported using butyric acid as a substrate, which has the same function with butyrate [70]. Alpha ketoglutarate is an essential metabolite of the mitochondrial TCA cycle, which can activate mitochondrial autophagy to inhibit ROS production [71], thereby slowing down oxidative stress and lowering the expression of *IL-6* and *TNF-α* to inhibit chronic low-grade inflammatory response [72]. Moreover, it contributes towards reducing the activity of myosin light-chain kinase (MLCK), enhancing the concentration of the tight junction (TJ) protein, regulating endoplasmic reticulum stress, and activating Wnt/β-. The proliferation and differentiation of intestinal stem cells using catenin drugs can restore barrier function [73,74]. Butyrate mediates DNA demethylation by up-regulating acetyl CoA and alpha ketoglutarate, thereby inhibiting the colon cancer transformation index [75]. Succinic semialdehyde (SSA) is the important metabolite of γ-aminobutyric acid (GABA) [76]. GABA can suppress immune responses by reducing T cell activity and levels of inflammatory mediators, making it a classic guardian of intestinal immune homeostasis [77]. It can also inhibit the signal transduction of hydrogen peroxide (H_2_O_2_) and superoxide anions to enhance the antioxidant mechanism in cells [78]. A previous study on the Nile tilapia also reported that supplementing GABA in the diet significantly increased the levels of protease, amylase, and lipase in the intestine [79]. Diacetyl plays a critical redox role in the cycle of oxygen to produce ROS in the body [80]. Butyric acid reduced the contents of diacetyl by the inhibition of diacetyl reductase [81]. Therefore, butanoate metabolism may have a positive effect on the antioxidant capacity, immune response, digestive enzyme activity, intestinal development, and intestinal barrier of Tibetan sheep jejunum.

The Spearman correlation between microbiome and metabolome showed that diacetyl was negatively correlated with *Methanobrevibacter*, *Actinobacteriota*, and *Bacillus.* It is speculated that butanoate metabolism in the RES, HMB and RES-HMB trial groups was related to the changes in *Methanobrevibacter*, *Actinobacteriota*, and *Bacillus*.

## 5. Conclusions

**Figure 9:**
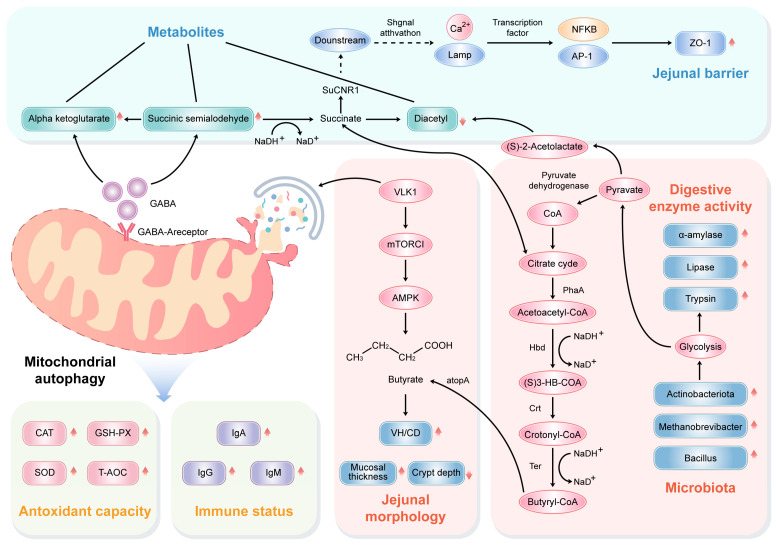
Schematic illustration demonstrating how feeding RES and (or) HMB addition improved butyrate concentration through modulating the microbial community and metabolism, thus contributing to jejunal morphology, antioxidant capacity, immune response, digestive enzyme activity and barrier function.

Our results indicated that the supplementation of RES and HMB alone or in combination efficiently improved butyrate formation, thus promoting intestinal morphological development and barrier function in the jejunum of Tibetan sheep. These changes may be closely related to the microbiota, and metabolites increased the contents of butyric acid in the jejunum. The proposed mechanism is represented in Figure 9. Therefore, these findings provide insights into detailed strategies for improving jejunal health in Tibetan sheep.

## Figures and Tables

**Table 1:** Dietary concentrate composition and nutrient levels (dry matter basis).

	Items	Content (%)
Ingredient	Corn	51.50
	Soybean meal	2.00
	Rapeseed meal	12.80
	Cottonseed meal	2.00
	Palm meal	25.00
	Nacl	1.00
	Limestone	1.00
	Baking soda	0.10
	Premix	4.60
	Total	100.00
Nutrient levels	Digestible energy (MJ/kg)	12.71
	Crude protein	14.27
	Ether extract	3.29
	Crude fiber	11.64
	Neutral detergent fiber	26.70
	Acid detergent fiber	19.97
	Ca	0.86
	P	0.40

(1) The premix provides 18 mg of Cu, 66 mg of Fe, 30 mg of Zn, 48 mg of Mn, 0.36 mg of Se, 0.6 mg of I, 0.24 mg of Co, 24,000 IU of VA, 4800 IU of VD, and 48 IU of VE per kilogram of feed. (2) Digestive energy is calculated, while the rest are measured values.

**Table 2:** Effects of supplementation with RES and HMB alone or in combination on the short-chain fatty acids (%).

Items	Groups				*p*-Value
	C	RES	HMB	RES-HMB	
Hexanoic acid	1.38 ± 0.62	0.76 ± 0.33	1.05 ± 0.37	1.25 ± 0.33	0.760
Isobutyric acid	2.70 ± 0.86	1.46 ± 1.08	0.64 ± 0.26	1.40 ± 0.53	0.343
Isovaleric acid	2.81 ± 2.41	3.50 ± 3.29	1.00 ± 0.73	0.53 ± 0.35	0.713
Butyric acid	2.29 ± 0.82 ^b^	5.44 ± 1.37 ^ab^	4.69 ± 0.54 ^b^	8.13 ± 1.11 ^a^	0.022
Propionic acid	5.39 ± 2.37	5.39 ± 1.75	4.55 ± 1.21	4.91 ± 1.46	0.982
Acetic acid	84.76 ± 5.55	83.23 ± 6.66	87.41 ± 1.25	82.77 ± 2.65	0.887
Valeric acid	0.68 ± 0.29	0.21 ± 0.04	0.66 ± 0.21	1.01 ± 0.35	0.240

^a,b^ means within a row with different subscripts when *p*-value < 0.05. The same below.

**Table 3:** Alpha diversity in the jejunum of Tibetan sheep among groups.

Items	Groups				*p*-Value
	C	RES	HMB	RES-HMB	
Ace	377.75 ± 12.85	473.95 ± 19.38	388.10 ± 40.33	407.83 ± 38.80	0.142
Chao 1	362.53 ± 11.34	461.73 ± 16.88	365.02 ± 37.74	397.04 ± 38.19	0.081
Shanon	2.16 ± 0.22	2.44 ± 0.13	1.80 ± 0.29	2.30 ± 0.31	0.382
Simpon	0.56 ± 0.07	0.61 ± 0.03	0.50 ± 0.08	0.58 ± 0.05	0.567
Sobs	288.33 ± 14.11	377.83 ± 17.45	287.33 ± 40.22	327.67 ± 42.13	0.167

## Data Availability

The datasets presented in this study can be found in online repositories. The names of the repository/repositories and accession number(s) can be found below: NCBI SRA (accession: PRJNA1127188). MetaboLights (accession: MTBLS10454).

## Supplementary Materials

The following supporting information can be downloaded at: https://www.mdpi.com/article/10.3390/antiox13080892/s1, Table S1, Primers used in qRT-PCR.

## References

[1] Shifflett D.E., Clayburgh D.R., Koutsouris A., Turner J.R., Hecht G.A. (2005). Enteropathogenic *E. coli* disrupts tight junction barrier function and structure in vivo. Lab. Investig..

[2] Ma Y., Yang X., Hua G., Deng X., Xia T., Li X., Feng D., Deng X. (2022). Contribution of gut microbiomes and their metabolomes to the performance of Dorper and Tan sheep. Front. Microbiol..

[3] Bi Y., Tu Y., Zhang N., Wang S., Zhang F., Suen G., Shao D., Li S., Diao Q. (2021). Multiomics analysis reveals the presence of a microbiome in the gut of fetal lambs. Gut.

[4] Su S., Wang L., Fu S., Zhao J., He X., Chen Q., Belobrajdic D.P., Yu C., Liu H., Wu H. (2022). Effects of oat (*Avena sativa* L.) hay diet supplementation on the intestinal microbiome and metabolome of Small-tail Han sheep. Front. Microbiol..

[5] Shoukry M.M., El-Nomeary Y., Salman F.M., Shakweer W.M.E. (2023). Improving the productive performance of growing lambs using prebiotic and probiotic as growth promoters. Trop. Anim. Health Prod..

[6] Shaito A., Posadino A.M., Younes N., Hasan H., Halabi S., Alhababi D., Al-Mohannadi A., Abdel-Rahman W.M., Eid A.H., Nasrallah G.K. (2020). Potential Adverse Effects of Resveratrol: A Literature Review. Int. J. Mol. Sci..

[7] Corrêa M.G., Absy S., Tenenbaum H., Ribeiro F.V., Cirano F.R., Casati M.Z., Pimentel S.P. (2019). Resveratrol attenuates oxidative stress during experimental periodontitis in rats exposed to cigarette smoke inhalation. J. Periodontal Res..

[8] Farrokhi E., Ghatreh-Samani K., Salehi-Vanani N., Mahmoodi A. (2018). The effect of resveratrol on expression of matrix metalloproteinase 9 and its tissue inhibitors in vascular smooth muscle cells. ARYA Atheroscler..

[9] Signorelli P., Ghidoni R. (2005). Resveratrol as an anticancer nutrient: Molecular basis, open questions and promises. J. Nutr. Biochem..

[10] Diao J., Wei J., Yan R., Fan G., Lin L., Chen M. (2019). Effects of resveratrol on regulation on UCP2 and cardiac function in diabetic rats. J. Physiol. Biochem..

[11] de la Lastra C.A., Villegas I. (2005). Resveratrol as an anti-inflammatory and anti-aging agent: Mechanisms and clinical implications. Mol. Nutr. Food. Res..

[12] Bhat K.P.L., Kosmeder J.W., Pezzuto J.M. (2001). Biological effects of resveratrol. Antioxid. Redox Signal..

[13] Hu Y., Chen D., Zheng P., Yu J., He J., Mao X., Yu B. (2019). The Bidirectional Interactions between Resveratrol and Gut Microbiota: An Insight into Oxidative Stress and Inflammatory Bowel Disease Therapy. Biomed. Res. Int..

[14] Larrosa M., Yañéz-Gascón M.J., Selma M.V., González-Sarrías A., Toti S., Cerón J.J., Tomás-Barberán F., Dolara P., Espín J.C. (2009). Effect of a low dose of dietary resveratrol on colon microbiota, inflammation and tissue damage in a DSS-induced colitis rat model. J. Agric. Food. Chem..

[15] Man A.W.C., Li H., Xia N. (2019). Resveratrol and the Interaction between Gut Microbiota and Arterial Remodelling. Nutrients.

[16] Holeček M. (2017). Beta-hydroxy-beta-methylbutyrate supplementation and skeletal muscle in healthy and muscle-wasting conditions. J. Cachexia Sarcopenia Muscle.

[17] Girón M.D., Vílchez J.D., Salto R., Manzano M., Sevillano N., Campos N., Argilés J.M., Rueda R., López-Pedrosa J.M. (2016). Conversion of leucine to β-hydroxy-β-methylbutyrate by α-keto isocaproate dioxygenase is required for a potent stimulation of protein synthesis in L6 rat myotubes. J. Cachexia Sarcopenia Muscle.

[18] Zhang S., Tang Z., Zheng C., Zhong Y., Zheng J., Duan G., Yin Y., Duan Y., Song Z. (2022). Dietary Beta-Hydroxy-Beta-Methyl Butyrate Supplementation Inhibits Hepatic Fat Deposition via Regulating Gut Microbiota in Broiler Chickens. Microorganisms.

[19] Duan Y., Zhong Y., Xiao H., Zheng C., Song B., Wang W., Guo Q., Li Y., Han H., Gao J. (2019). Gut microbiota mediates the protective effects of dietary β-hydroxy-β-methylbutyrate (HMB) against obesity induced by high-fat diets. FASEB J..

[20] Beaudart C., Rabenda V., Simmons M., Geerinck A., Araujo De Carvalho I., Reginster J.Y., Amuthavalli Thiyagarajan J., Bruyère O. (2018). Effects of Protein, Essential Amino Acids, B-Hydroxy B-Methylbutyrate, Creatine, Dehydroepiandrosterone and Fatty Acid Supplementation on Muscle Mass, Muscle Strength and Physical Performance in Older People Aged 60 Years and Over. A Systematic Review on the Literature. J. Nutr. Health Aging.

[21] Zhu K.A., Zhang Y., Zhang F.S., Wu Z.L., Su Q., Hou S.Z., Gui L.S. (2024). The Effects of Dietary Resveratrol and β-Hydroxy-β-Methylbutyric Acid Supplementation at Two Protein Levels on the Ruminal Microbiome and Metabolome of Tibetan Sheep. Agriculture.

[22] Sies H. (2015). Oxidative stress: A concept in redox biology and medicine. Redox Biol..

[23] Yuan D., Hussain T., Tan B., Liu Y., Ji P., Yin Y. (2017). The Evaluation of Antioxidant and Anti-Inflammatory Effects of Eucommia ulmoides Flavones Using Diquat-Challenged Piglet Models. Oxid. Med. Cell. Longev..

[24] Ding X., Cai C., Jia R., Bai S., Zeng Q., Mao X., Xu S., Zhang K., Wang J. (2022). Dietary resveratrol improved production performance, egg quality, and intestinal health of laying hens under oxidative stress. Poult. Sci..

[25] Yang C., Luo P., Chen S.J., Deng Z.C., Fu X.L., Xu D.N., Tian Y.B., Huang Y.M., Liu W.J. (2021). Resveratrol sustains intestinal barrier integrity, improves antioxidant capacity, and alleviates inflammation in the jejunum of ducks exposed to acute heat stress. Poult. Sci..

[26] Arazi H., Hosseini Z., Asadi A., Ramirez-Campillo R., Suzuki K. (2019). β-Hydroxy-β-Methylbutyrate Free Acid Attenuates Oxidative Stress Induced by a Single Bout of Plyometric Exercise. Front. Physiol..

[27] Peake J.M., Suzuki K., Coombes J.S. (2007). The influence of antioxidant supplementation on markers of inflammation and the relationship to oxidative stress after exercise. J. Nutr. Biochem..

[28] Hussain T., Tan B., Yin Y., Blachier F., Tossou M.C., Rahu N. (2016). Oxidative Stress and Inflammation: What Polyphenols Can Do for Us?. Oxid. Med. Cell. Longev..

[29] Luckose F., Pandey M.C., Radhakrishna K. (2015). Effects of amino acid derivatives on physical, mental, and physiological activities. Crit. Rev. Food Sci. Nutr..

[30] Ruocco C., Segala A., Valerio A., Nisoli E. (2021). Essential amino acid formulations to prevent mitochondrial dysfunction and oxidative stress. Curr. Opin. Clin. Nutr. Metab. Care.

[31] Gan Z., Wei W., Li Y., Wu J., Zhao Y., Zhang L., Wang T., Zhong X. (2019). Curcumin and Resveratrol Regulate Intestinal Bacteria and Alleviate Intestinal Inflammation in Weaned Piglets. Molecules.

[32] Fu Q., Cui Q., Yang Y., Zhao X., Song X., Wang G., Bai L., Chen S., Tian Y., Zou Y. (2018). Effect of Resveratrol Dry Suspension on Immune Function of Piglets. Evid. Based Complement. Altern. Med..

[33] Adams A.A., Siard M.H., Reedy S.E., Stewart C., Betancourt A., Sanz M.G., Horohov D.W. (2013). Identifying the role of a “caloric restriction mimetic”, resveratrol, in Equine Metabolic Syndrome and its implications for targeted therapy. J. Equine Vet. Sci..

[34] Miyake S., Ogo A., Kubota H., Teramoto F., Hirai T. (2019). β-Hydroxy-β-methylbutyrate Suppresses NF-ĸB Activation and IL-6 Production in TE-1 Cancer Cells. Vivo.

[35] Smith H.J., Wyke S.M., Tisdale M.J. (2004). Mechanism of the attenuation of proteolysis-inducing factor stimulated protein degradation in muscle by beta-hydroxy-beta-methylbutyrate. Cancer Res..

[36] Fraga A.Z., Campos P., Hauschild L., Chalvon-Demersay T., Beaumont M., Le Floc’h N. (2023). A blend of functional amino acids and grape polyphenols improves the pig capacity to cope with an inflammatory challenge caused by poor hygiene of housing conditions. BMC Vet. Res..

[37] Yvon S., Beaumont M., Dayonnet A., Eutamène H., Lambert W., Tondereau V., Chalvon-Demersay T., Belloir P., Paës C. (2024). Effect of diet supplemented with functional amino acids and polyphenols on gut health in broilers subjected to a corticosterone-induced stress. Sci. Rep..

[38] Afzali-Kordmahalleh A., Meshkini S. (2023). Effects of dietary resveratrol supplementation on digestive enzymes activities and serum biochemistry of rainbow trout (*Oncorhynchus mykiss*). Vet. Res. Forum..

[39] Yang S., Xu W., Feng L., Zhang C., Yan C., Zhang J., Lai J., Yan T., He Z., Du X. (2022). Resveratrol Improves the Digestive Ability and the Intestinal Health of Siberian Sturgeon. Int. J. Mol. Sci..

[40] Foye O.T., Ferket P.R., Uni Z. (2007). The effects of in ovo feeding arginine, beta-hydroxy-beta-methyl-butyrate, and protein on jejunal digestive and absorptive activity in embryonic and neonatal turkey poults. Poult.

[41] Zhang J., Chai X., Zhao F., Hou G., Meng Q. (2022). Food Applications and Potential Health Benefits of Hawthorn. Foods.

[42] Kwon O., Han T.S., Son M.Y. (2020). Intestinal Morphogenesis in Development, Regeneration, and Disease: The Potential Utility of Intestinal Organoids for Studying Compartmentalization of the Crypt-Villus Structure. Front. Cell Dev. Biol..

[43] Wilson F.D., Cummings T.S., Barbosa T.M., Williams C.J., Gerard P.D., Peebles E.D. (2018). Comparison of two methods for determination of intestinal villus to crypt ratios and documentation of early age-associated ratio changes in broiler chickens. Poult. Sci..

[44] Gao Y., Meng L., Liu H., Wang J., Zheng N. (2020). The Compromised Intestinal Barrier Induced by Mycotoxins. Toxins.

[45] Chen X., Zeng Z., Huang Z., Chen D., He J., Chen H., Yu B., Yu J., Luo J., Luo Y. (2021). Effects of dietary resveratrol supplementation on immunity, antioxidative capacity and intestinal barrier function in weaning piglets. Anim. Biotechnol..

[46] Liu L., Fu C., Yan M., Xie H., Li S., Yu Q., He S., He J. (2016). Resveratrol modulates intestinal morphology and HSP70/90, NF-κB and EGF expression in the jejunal mucosa of black-boned chickens on exposure to circular heat stress. Food Funct..

[47] Suad K.A., Al-Shamire J.S.H., Dhyaa A.A. (2018). Histological and biochemical evaluation of supplementing broiler diet with β-hydroxy-methyl butyrate calcium (β-HMB-Ca). Iran. J. Vet. Res..

[48] Zheng C., Song B., Duan Y., Zhong Y., Yan Z., Zhang S., Li F. (2020). Dietary β-hydroxy-β-methylbutyrate improves intestinal function in weaned piglets after lipopolysaccharide challenge. Nutrition.

[49] Johansson M.E., Phillipson M., Petersson J., Velcich A., Holm L., Hansson G.C. (2008). The inner of the two Muc2 mucin-dependent mucus layers in colon is devoid of bacteria. Proc. Natl. Acad. Sci. USA.

[50] Fang Q., Wang J.F., Zha X.Q., Cui S.H., Cao L., Luo J.P. (2015). Immunomodulatory activity on macrophage of a purified polysaccharide extracted from Laminaria japonica. Carbohydr. Polym..

[51] Wang P., Wang J., Li D., Ke W., Chen F., Hu X. (2020). Targeting the gut microbiota with resveratrol: A demonstration of novel evidence for the management of hepatic steatosis. J. Nutr. Biochem..

[52] Dou Z., Rong X., Zhao E., Zhang L., Lv Y. (2019). Neuroprotection of Resveratrol Against Focal Cerebral Ischemia/Reperfusion Injury in Mice Through a Mechanism Targeting Gut-Brain Axis. Cell. Mol. Neurobiol..

[53] Zhou J.Y., Wang Z., Zhang S.W., Lin H.L., Gao C.Q., Zhao J.C., Yang C., Wang X.Q. (2019). Methionine and Its Hydroxyl Analogues Improve Stem Cell Activity to Eliminate Deoxynivalenol-Induced Intestinal Injury by Reactivating Wnt/β-Catenin Signaling. J. Agric. Food Chem..

[54] Pan F.Y., Wu P., Feng L., Jiang W.D., Kuang S.Y., Tang L., Tang W.N., Zhang Y.A., Zhou X.Q., Liu Y. (2017). Methionine hydroxy analogue improves intestinal immunological and physical barrier function in young grass carp (*Ctenopharyngodon idella*). Fish Shellfish Immunol..

[55] Zhang D., Jian Y.P., Zhang Y.N., Li Y., Gu L.T., Sun H.H., Liu M.D., Zhou H.L., Wang Y.S., Xu Z.X. (2023). Short-chain fatty acids in diseases. Cell Commun. Signal..

[56] Alrafas H.R., Busbee P.B., Nagarkatti M., Nagarkatti P.S. (2019). Resveratrol modulates the gut microbiota to prevent murine colitis development through induction of Tregs and suppression of Th17 cells. J. Leukoc. Biol..

[57] Baghbanzadeh-Nobari B., Taghizadeh A., Khorvash M., Parnian-Khajehdizaj F., Maloney S.K., Hashemzadeh-Cigari F., Ghaffari A.H. (2017). Digestibility, ruminal fermentation, blood metabolites and antioxidant status in ewes supplemented with DL-methionine or hydroxy-4 (methylthio) butanoic acid isopropyl ester. J. Anim. Physiol. Anim. Nutr..

[58] Hamer H.M., Jonkers D.M., Bast A., Vanhoutvin S.A., Fischer M.A., Kodde A., Troost F.J., Venema K., Brummer R.J. (2009). Butyrate modulates oxidative stress in the colonic mucosa of healthy humans. Clin. Nutr..

[59] Li X., Wang C., Zhu J., Lin Q., Yu M., Wen J., Feng J., Hu C. (2022). Sodium Butyrate Ameliorates Oxidative Stress-Induced Intestinal Epithelium Barrier Injury and Mitochondrial Damage through AMPK-Mitophagy Pathway. Oxid. Med. Cell. Longev..

[60] Dang G., Wu W., Zhang H., Everaert N. (2021). A new paradigm for a new simple chemical: Butyrate & immune regulation. Food Funct..

[61] Binda C., Lopetuso L.R., Rizzatti G., Gibiino G., Cennamo V., Gasbarrini A. (2018). Actinobacteria: A relevant minority for the maintenance of gut homeostasis. Dig. Liver Dis..

[62] Valliappan K., Sun W., Li Z. (2014). Marine actinobacteria associated with marine organisms and their potentials in producing pharmaceutical natural products. Appl. Microbiol. Biotechnol..

[63] Frontiers Production Office (2020). Erratum: Microbiota of the Gut-Lymph Node Axis: Depletion of Mucosa-Associated Segmented Filamentous Bacteria and Enrichment of Methanobrevibacter by Colistin Sulfate and Linco-Spectin in Pigs. Front. Microbiol..

[64] Fuller M., Priyadarshini M., Gibbons S.M., Angueira A.R., Brodsky M., Hayes M.G., Kovatcheva-Datchary P., Bäckhed F., Gilbert J.A., Lowe W.L. (2015). The short-chain fatty acid receptor, FFA2, contributes to gestational glucose homeostasis. Am. J. Physiol. Endocrinol. Metab..

[65] Yan M., Yin W., Fang X., Guo J., Shi H. (2016). Characteristics of a water-forming NADH oxidase from Methanobrevibacter smithii, an archaeon in the human gut. Biosci. Rep..

[66] Orgler E., Baumgartner M., Duller S., Kumptisch C., Hausmann B., Moser D., Khare V., Lang M., Köcher T., Frick A. (2024). Archaea influence composition of endoscopically visible ileocolonic biofilms. Gut Microbes.

[67] Chen Y.W., Yu Y.H. (2023). Differential effects of Bacillus subtilis- and Bacillus licheniformis-fermented products on growth performance, intestinal morphology, intestinal antioxidant and barrier function gene expression, cecal microbiota community, and microbial carbohydrate-active enzyme composition in broilers. Poult. Sci..

[68] Chang W.Y., Yu Y.H. (2022). Effect of Bacillus species-fermented products and essential oils on growth performance, gut morphology, cecal short-chain fatty acid levels, and microbiota community in broilers. Poult. Sci..

[69] Cheng Y.H., Horng Y.B., Dybus A., Yu Y.H. (2021). Bacillus licheniformis-Fermented Products Improve Growth Performance and Intestinal Gut Morphology in Broilers under *Clostridium perfringens* Challenge. J. Poult. Sci..

[70] Gonçalves P., Martel F. (2013). Butyrate and colorectal cancer: The role of butyrate transport. Curr. Drug Metab..

[71] Averina O.A., Permyakov O.A., Emelianova M.A., Grigoryeva O.O., Gulyaev M.V., Pavlova O.S., Mariasina S.S., Frolova O.Y., Kurkina M.V., Baydakova G.V. (2023). Mitochondrial peptide Mtln contributes to oxidative metabolism in mice. Biochimie.

[72] Liu L., Zhang W., Liu T., Tan Y., Chen C., Zhao J., Geng H., Ma C. (2023). The physiological metabolite α-ketoglutarate ameliorates osteoarthritis by regulating mitophagy and oxidative stress. Redox Biol..

[73] Bravo Iniguez A., Du M., Zhu M.J. (2024). α-Ketoglutarate for Preventing and Managing Intestinal Epithelial Dysfunction. Adv. Nutr..

[74] Si X., Song Z., Liu N., Jia H., Liu H., Wu Z. (2022). α-Ketoglutarate Restores Intestinal Barrier Function through Promoting Intestinal Stem Cells-Mediated Epithelial Regeneration in Colitis. J. Agric. Food Chem..

[75] Sun X., Zhu M.J. (2018). Butyrate Inhibits Indices of Colorectal Carcinogenesis via Enhancing α-Ketoglutarate-Dependent DNA Demethylation of Mismatch Repair Genes. Mol. Nutr. Food Res..

[76] van Bemmelen F.J., Schouten M.J., Fekkes D., Bruinvels J. (1985). Succinic semialdehyde as a substrate for the formation of gamma-aminobutyric acid. J. Neurochem..

[77] Auteri M., Zizzo M.G., Serio R. (2015). GABA and GABA receptors in the gastrointestinal tract: From motility to inflammation. Pharmacol. Res..

[78] Beltrán González A.N., López Pazos M.I., Calvo D.J. (2020). Reactive Oxygen Species in the Regulation of the GABA Mediated Inhibitory Neurotransmission. Neuroscience.

[79] Ruenkoed S., Nontasan S., Phudkliang J., Phudinsai P., Pongtanalert P., Panprommin D., Mongkolwit K., Wangkahart E. (2023). Effect of dietary gamma aminobutyric acid (GABA) modulated the growth performance, immune and antioxidant capacity, digestive enzymes, intestinal histology and gene expression of Nile tilapia (*Oreochromisniloticus*). Fish Shellfish Immunol..

[80] Kovacic P., Cooksy A.L. (2005). Role of diacetyl metabolite in alcohol toxicity and addiction via electron transfer and oxidative stress. Arch. Toxicol..

[81] Morris J.B., Hubbs A.F. (2009). Inhalation dosimetry of diacetyl and butyric acid, two components of butter flavoring vapors. Toxicol. Sci..

