# Supplementary material for: Multi-Omics Revealed Resveratrol and β-Hydroxy-β-methyl Butyric Acid Alone or in Combination Improved the Jejunal Function in Tibetan Sheep
Source: Antioxidants (Basel). 2024 Jul 24;13(8):892. doi: 10.3390/antiox13080892 (PMC11351831; doi:10.3390/antiox13080892)
Supplement: Supplementary file 1 [file antioxidants-13-00892-s001.zip › antioxidants-3095915-supplementary.pdf]

Table S1 Primers used in qRT-PCR

Name	Primer sequence (5'-3')	Tm (℃)	Product length
claudin-1	F-CCTGCTGTGCTGCTCCTGTC	61.6	75bp
	R-GAAGGTGCTGGCTTGGGATAGG	61.4	
occludin	F-CGAGAAGCGACCGTATCCAGAG	61.4	129bp
	R-TCCAAGTTACCACTGCTGCTGTAG	59.6	
muc-2	F-ACGACTCCTACGCCCTCCTG	61.6	130bp
	R-ACGCTGCCATCCGACTTGAAG	59.5	
ZO-1	F-GGGCAAGTTAAAGATGGTGGTTCAG	59.6	93bp
	R-GAGGCGTCAGCAGAGTGGATG	61.5	
TNF-α	F-ACGGCGTGGAGCTGAAAGAC	59.5	79bp
	R-CTGAAGAGGACCTGCGAGTAGATG	61.3	
IL-6	F-TCTAATAACCACTCCAGCCACACAC	59.6	77bp
	R-TTGCGTTCTTTACCCACTCGTTTG	57.9	
IL-1β	F-GGCAGGCAGTGTCGGTCATC	61.6	83bp
	R-CCTCAGGTCATCATCACGGAAGAC	61.3	
IL-10	F-AATGAAGGACCAACTGAACAGCATG	57.9	87bp
	R-TCCGACAAGGCTTGGCAACC	59.5	
β-Actin	F-AGCAAGCGTGGCATCCTAACC	59.5	77bp
	R-ATCTTCTCCATGTCGTCCCAGTTG	59.6	
